# Isatuximab for Delayed Red Cell Engraftment after Allogeneic Hematopoietic Cell Transplantation

**DOI:** 10.1155/2024/5790011

**Published:** 2024-08-30

**Authors:** Mary Nauffal, Stephen Eng, Andrew Lin, Alexander Chan, Kathryn Mazzerella, Sergio Giralt, Miguel-Angel Perales, Boglarka Gyurkocza

**Affiliations:** ^1^ Department of Pharmacy Memorial Sloan Kettering Cancer Center, New York, NY, USA; ^2^ Department of Pathology Hematopathology Service Memorial Sloan Kettering Cancer Center, New York, NY, USA; ^3^ Department of Advanced Practice Providers Memorial Sloan Kettering Cancer Center, New York, NY, USA; ^4^ Department of Medicine Adult Bone Marrow Transplantation Service Memorial Sloan Kettering Cancer Center, New York, NY, USA; ^5^ Department of Medicine Weill Cornell Medical College of Cornell University, New York, NY, USA

## Abstract

Isatuximab is an IgG1*κ*-derived monoclonal antibody against CD38 approved for the treatment of adult patients with multiple myeloma. Here we describe the successful treatment of a therapy-refractory pure red cell aplasia case following ABO-mismatched allogeneic stem cell transplantation with isatuximab. Our patient was a 75-year-old female with acute myeloid leukemia who received an HLA-B antigen mismatched, unrelated peripheral blood stem cell transplant with a major ABO incompatibility (blood group A+ in the donor and blood group O+ in the recipient). The patient developed persistent red cell aplasia and anti-A antibodies for more than 500 days from transplant. She received therapy with rituximab, bortezomib, prednisone, and darbepoetin alfa with partial to no response. After repeated insurance denials for daratumumab, isatuximab was obtained from the manufacturer through their CareASSIST program. Following the completion of 2 cycles of isatuximab (8 doses), significant and sustained red cell recovery was observed.

## 1. Introduction

Pure red cell aplasia (PRCA) is a potential complication following allogeneic hematopoietic cell transplantation (allo-HCT) due to major or bidirectional ABO incompatibility. It is characterized by severe anemia with reticulocytopenia after excluding other causes for anemia including infections, hemolysis, disease relapse, or drug toxicity [1]. Risk factors include HLA-matching, high recipient pretransplant isohemagglutinins, utilization of reduced-intensity conditioning (RIC) or non-myeloablative (NMA) regimens, and absence of graft vs. host disease (GVHD) [2]. While cases of PRCA following allo-HCT resolve spontaneously, 30–40% of patients require additional therapies with immunosuppressive agents, particularly if elevated anti-donor isohemagglutinins persist beyond 60 days post-transplant [1]. Treatments include corticosteroids, recombinant human erythropoietin, antithymocyte globulin, cellular therapies, and monoclonal antibodies such as rituximab (anti-CD20) and daratumumab (anti-CD38) [3].

Isatuximab is an IgG1*κ*-derived monoclonal antibody that binds CD38 expressed on the surface of hematopoietic and tumor cells [4]. Due to its similar mechanism of action with daratumumab, we hypothesized that administering an alternative anti-CD38 monoclonal antibody would allow for red cell recovery. Herein, we describe a case of refractory PRCA post-allo-HCT that was successfully managed with isatuximab.

## 2. Case Presentation

**Figure 1:**
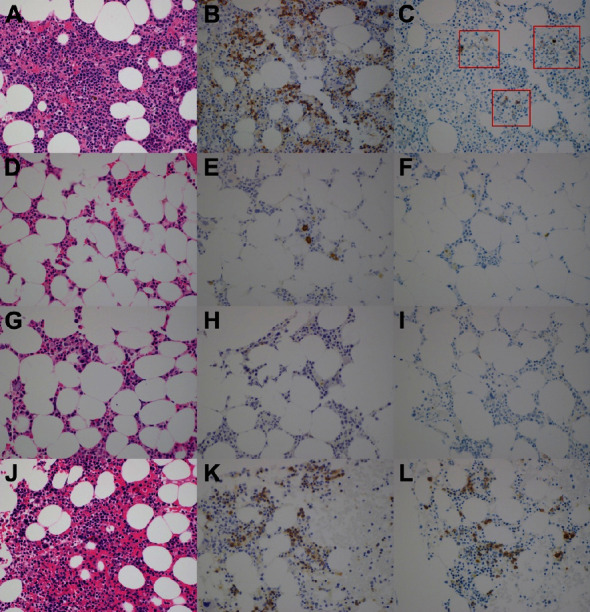
Histologic images of the patient's bone marrow sample. (A) Pretransplant bone marrow H&E stained histologic image shows a cellular bone marrow with maturing trilineage hematopoiesis. (B) CD71 immunohistochemical stain highlights an appropriate number and proportion of erythroid precursors. (C) E-cadherin immunohistochemical stain weakly stains early erythroid precursors (red boxes). (D) Day 66 post-transplant bone marrow H&E shows a hypocellular bone marrow with markedly reduced erythroid elements. (E) CD71 shows very rare erythroid elements. (F) E-cadherin is essentially negative. (G) Bone marrow H&E stained histologic image after last dose of bortezomib shows a persistently hypocellular marrow. (H, I) CD71 and E-cadherin immunohistochemical stains show that erythroid precursors are essentially absent. (J) H&E image of bone marrow after multiple doses of isatuximab shows a normocellular bone marrow with maturing trilineage hematopoiesis. (K) CD71 highlights erythroid precursors which are once again seen in normal proportion and numbers. (L) E-cadherin shows early erythroid precursors. All images shown at 400x magnification.

A 75-year-old woman was referred to the bone marrow transplantation clinic for consideration of an allo-HCT for high-risk acute myeloid leukemia (AML). She achieved a complete response with minimal residual disease negativity following induction and consolidation chemotherapy. The best donor identified was an HLA-B mismatched donor with a major ABO incompatibility. The patient and donor had blood type O and A, respectively. She received RIC consisting of cyclophosphamide, fludarabine, and low-dose total body irradiation (2 Gy) followed by a peripheral blood stem cell graft. GVHD prophylaxis included post-transplant cyclophosphamide, sirolimus, and mycophenolate mofetil (MMF). Neutrophil and platelet engraftment occurred 15 and 22 days following HCT, respectively. Day + 66 marrow biopsy showed no evidence of AML, 100% donor chimerism, and marked erythroid hypoplasia (<1%). Immunohistochemical staining for CD71 and E-cadherin confirmed absence of erythroid precursors (Figure 1). No viral nuclear inclusions suggestive of parvovirus infection were seen, and immunohistochemistry for parvovirus was also negative. MMF and sirolimus were discontinued on day + 55 and day + 356, respectively.

**Figure 2:**
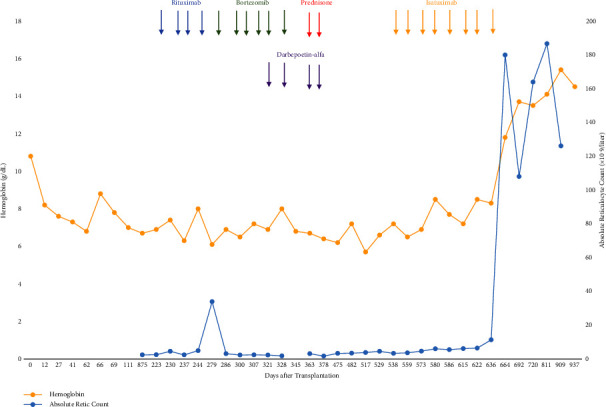
Treatment outcomes of therapies for pure red cell aplasia. Shown are the hemoglobin and reticulocyte count after therapy with rituximab, bortezomib, darbepoetin alfa, prednisone, and isatuximab.

Her post-transplant course was complicated by persistent anemia requiring frequent red cell transfusions. Hemolysis studies showed elevated anti-A and anti-B antibody titers. Rituximab was initiated but she remained transfusion dependent. Subsequently, the patient received bortezomib, darbepoetin, and low-dose prednisone, yet no response was observed (Figure 2). Daratumumab was denied by insurance and consequently, isatuximab was obtained via the manufacturer's CareASSIST program and initiated at a dose of 10 mg/kg intravenously weekly for 4 doses followed by every 2 weeks. One month prior to isatuximab initiation, elevated liver enzymes (alanine transaminase (ALT) levels greater than 1.5 times the upper limit of normal (ULN)) coupled with high ferritin levels showed marked hepatic hemosiderosis on the liver magnetic resonance imaging. Treatment of iron overload with deferasirox was not initiated due to insurance denial for coverage. Her ALT stabilized to 0.5 times the ULN following initiation of isatuximab but her ferritin levels remained elevated throughout her treatment course. She was not a candidate for phlebotomies due to severe anemia and ongoing PRCA. After 4 doses of isatuximab, erythroid precursors were present but markedly reduced and following the eighth dose of isatuximab, an increase in the number of erythroid precursors with left shifted maturation was seen on her bone marrow biopsy. At that time, she also became transfusion independent, and her hemoglobin was normal. As of 30 months following treatment with isatuximab, she remains transfusion independent with normal levels of hemoglobin (15.9 g/dL).

Adverse events were limited to grade 3 neutropenia, with an absolute neutrophil count of 100 neutrophils/microL, lasting for 1 week and leading to the delayed administration of the sixth dose of isatuximab by a week. Her brief neutropenia episode was successfully managed with 1 dose of colony-stimulating growth factor. She did not develop any infections or GVHD during her treatment.

## 3. Discussion

The pathophysiology of PRCA is not fully elucidated but is thought to involve the persistence of the recipient's anti-A or anti-B antibodies against donor red blood cells [2]. The incidence of PRCA following major ABO incompatibility is high with RIC or NMA regimens compared to myeloablative conditioning regimens due to prolonged persistence of both recipient and donor hematopoiesis (mixed chimerism) [2]. In addition, it is higher in patients with blood type O receiving a transplant from a donor with blood type A due to increased antigenicity of A antigens as compared to B antigens [2]. The patient described here had persistent elevations of anti-donor antibodies for more than 500 days after transplantation making it the most likely primary cause of PRCA.

We administered erythropoietin, corticosteroids, and rituximab with no response [5]. Our outcome aligns with the findings of a large retrospective study by Longval and colleagues who did not find a significant benefit from these agents and hypothesized that PRCA may be associated with plasma cell chimerism and to a less extent with mature B lymphocytes that harbor CD20 [2]. Subsequently, bortezomib was administered based on its indirect impact on plasma cells but resulted in a partial response [5].

The association of PRCA with residual elevated isohemagglutinins producing plasma cells makes CD38 a suitable target, as it is overexpressed on the surface of plasma cells and may eliminate the plasma cell clone responsible for delayed erythroid engraftment [2]. Based on successful reports of daratumumab for PRCA post-allo-HCT, isatuximab was considered as an alternative anti-CD38 monoclonal antibody [3]. While they share similar mechanisms of action, isatuximab and daratumumab monoclonal antibody structures have different amino acid sequences within the complementarity determining regions of the heavy chains leading to different binding modes to CD38 epitopes which in turn contribute to the distinct mode of action. Isatuximab's distinguished feature is its ability to inhibit CD38 ectoenzymatic activity as well as directly induce apoptosis while daratumumab requires to be combined with cross-linking agents to induce cell death [6, 7]. Isatuximab triggers both the caspase-dependent apoptotic pathway and the lysosome-mediated cell death pathway [4]. These differences may explain the possibly higher hematological toxicity with isatuximab over daratumumab. Our patient experienced a short period of neutropenia that was successfully managed with colony-stimulating growth factor support as per the manufacturer's recommendation for improving neutrophil count.

Isatuximab was effective and well tolerated in this case of severe refractory PRCA for more than 500 days post-transplant. The delayed response could be explained by her prolonged duration of anemia and large number of packed RBC transfused (50 units of blood transfusions). We can safely presume that the favorable outcome was primarily due to isatuximab as it was administered 9 months from rituximab, allowing for a sufficient wash out period between the two monoclonal antibodies. No infections or GVHD was observed following 14 months after treatment completion with isatuximab.

To our knowledge, this is the first case report to describe the complete remission of refractory PRCA following allo-HCT with isatuximab and it seems to be a promising agent. Prospective randomized studies are warranted to explore the role of isatuximab for refractory PRCA.

## Data Availability

Data are available on request from the authors.

## Additional Points

*What Is the New Aspect of Your Work?* This is the first successful case report on the use of isatuximab for the management of delayed pure red cell aplasia following allogeneic hematopoietic cell transplantation. *What Is the Central Finding of Your Work?* Isatuximab, a newer CD38-directed antibody, successfully restored red cell engraftment after more than 500 days from allogeneic hematopoietic cell transplantation. *What Is (or Could Be) the Specific Clinical Relevance of Your Work?* Isatuximab represents a new alternative for the management of delayed red blood cell engraftment following allogeneic hematopoietic stem cell transplantation.
